# Measuring the ATLAS ITk pixel detector material via multiple scattering of positrons at the CERN PS

**DOI:** 10.1140/epjc/s10052-025-14092-2

**Published:** 2025-04-03

**Authors:** Simon Florian Koch, Brian Moser, Antonín Lindner, Valerio Dao, Ignacio Asensi, Daniela Bortoletto, Marianne Brekkum, Florian Dachs, Hans Ludwig Joos, Milou van Rijnbach, Abhishek Sharma, Ismet Siral, Carlos Solans, Yingjie Wei

**Affiliations:** 1 Department of Physics, Oxford University, Oxford, UK; 2 CERN, Geneva, Switzerland; 3 II. Physikalisches Institut, Georg-August-Universität Göttingen, Göttingen, Germany; 4 Department of Physics, University of Oslo, Oslo, Norway; 5 Departments of Physics and Astronomy, Stony Brook University, Stony Brook, NY USA

## Abstract

The ITk is a new silicon tracker for the ATLAS experiment designed to increase detector resolution, readout capacity, and radiation hardness, in preparation for the larger number of simultaneous proton–proton interactions at the High Luminosity LHC. This paper presents the first direct measurement of the material budget of an ATLAS ITk pixel module, performed at a testbeam at the CERN Proton Synchrotron via the multiple scattering of low energy positrons within the module volume. Using a four plane telescope of thin monolithic pixel detectors from the MALTA Collaboration, scattering datasets were recorded at a beam energy of $1.2\,\text {GeV}$. Kink angle distributions were extracted from tracks derived with and without information from the ITk pixel module, and were fit to extract the RMS scattering angle, which was converted to a fractional radiation length $x/X_0$. The average $x/X_0$ across the module was measured as $[0.89 \pm 0.01 \text { (resolution)} \pm 0.01 \text { (subtraction)} \,\,\pm \,\, 0.08 \text {~(beam momentum band)}]\%$, which agrees within uncertainties with an estimate of $0.88\%$ derived from material component expectations.

## Introduction

The High Luminosity upgrade of the LHC (HL-LHC) will increase the instantaneous luminosity of the machine up to $7.5 \times 10^{34}\,\text {cm}^{-2}\,\text {s}^{-1}$, facilitating an order of magnitude increase in the delivered proton–proton collision data [1]. This increase in instantaneous luminosity will proportionally increase data rates and radiation damage in the ATLAS detector. The entire inner detector system will be replaced by an all-silicon tracker, the Inner Tracker (ITk) [2–4], to keep excellent physics performance in this harsh environment. The ITk will improve over the currently installed tracker by having a higher granularity, an improved radiation hardness and an increased coverage in the forward direction. Requirements on the tracking and vertexing performance of the ITk mandate the detector to have a lower material budget, especially for the innermost layers, which will feature silicon pixel detectors. Accurate knowledge of this material content is paramount to ensure the correctness of performance estimations and in turn the expected physics output of the experiment. It is also a key incredient to the simulations that will later on be compared to data in the measurements themselves. This paper presents a first measurement of the fractional radiation length of an ITk pixel module via the multiple scattering of $1.2\,\text {GeV}$ positrons at the T9 beamline of the CERN Proton Synchrotron. Alongside the measurement methodology, the design and construction of the purpose-built MONSTAR beam telescope will be discussed, as will the analysis method and results.

**Fig. 1:**
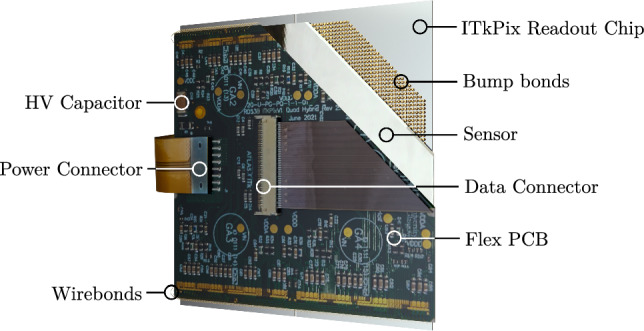
Annotated render of an ITk pixel detector quad module, populated with four ITkPix v1.1 ROCs and quad flex PCB v2.4, with a cutaway revealing the main layers. The high voltage (HV) capacitor and the data and power connectors have been highlighted as these are substantial contributions to the material budget of the module

The pixel modules used in the ITk vary in sensor technology depending on their position in the detector. The innermost layer uses 3D sensors, while the outer layers use planar sensors with varying thickness. A hybrid pixel module design is used, with sensors bump bonded to readout chips (ROCs), which are in turn wire bonded to a printed circuit board (PCB) with connectors to the outside world. The ITkPix ROCs were developed by the RD53 Collaboration [5] and varying pixel pitches are used within the sensor layout, depending again on the location of the module in the detector. The module used in this measurement is a quad module designed for the Outer Barrel part of the ITk pixel detector, consisting of 4 ROCs in a $2 \times 2$ matrix bump bonded to a single silicon sensor. A cutaway view of the module is shown in Fig. 1. Especially the populated PCB with its connectors, pads and surface mount devices (SMDs) makes the module an ideal target for a spatially resolved fractional radiation length measurement. This is further exacerbated by the fact that for many of these components it is difficult to get an accurate estimate of their radiation length.

Multiple (Coulomb) scattering can be described within Molière theory [6], predicting the scattering angle distribution to be composed of three analytic terms: a Gaussian core analogous to the central limit of a large number of soft scatters, a transition to the Rutherford formula for large angle scatters, and a correction. The root mean square (RMS) of the Gaussian core of the scatter angle projected onto a reference plane parallel to the incident direction can be related to the fractional radiation length $x/X_{0}$ with the Lynch & Dahl revision of the Highland formula^1^ [7, 8] as

1 $$\begin{aligned} \begin{aligned}&\theta ^{\text {RMS}}_{\text {Highland}} = \frac{13.6\,\text {MeV}}{\beta c p} z \sqrt{x/X_{0}}\left[ 1 + 0.038\ln \left( \frac{xz^2}{X_{0}\beta ^2}\right) \right] \text {.} \end{aligned} \end{aligned}$$

Here, $\beta c$, *p* and *z* are the speed, momentum, and charge number of the particle that undergoes multiple scattering. This formula can be analytically inverted to calculate the fractional radiation length from the measured distribution of scatter angles. An alternative description by Frühwirth and Regler [9], which derives from a fit to a large convolution of single scattering distributions, improves over the Highland formalism by adding one additional parameter in the fit to derive the Gaussian core expression.

Multiple scattering based radiation length measurements at testbeams have been performed before, for example using a six plane MIMOSA telescope at the DESY II testbeam facility or similar beamlines [10–12], but generally focussed on relatively thick subjects in relation to a pixel module, and did not attempt to image instrumented detectors. A recent measurement by the Tracker Group of the CMS Collaboration imaged an upgrade pixel module at the PSI PiE1 beamline [13]. This work builds on the previous to design and build a four plane telescope based on MALTA detectors, and perform the first measurement of the radiation length of an entire ITk pixel module with sub-mm resolution and ${\mathcal {O}}(10\%)$ uncertainties. We present results for the fractional radiation length obtained with Highland’s formalism and the improved Frühwirth–Regler description, as well as from a parametric fit to GEANT4 based simulation [14].

## Experimental setup

**Fig. 2:**
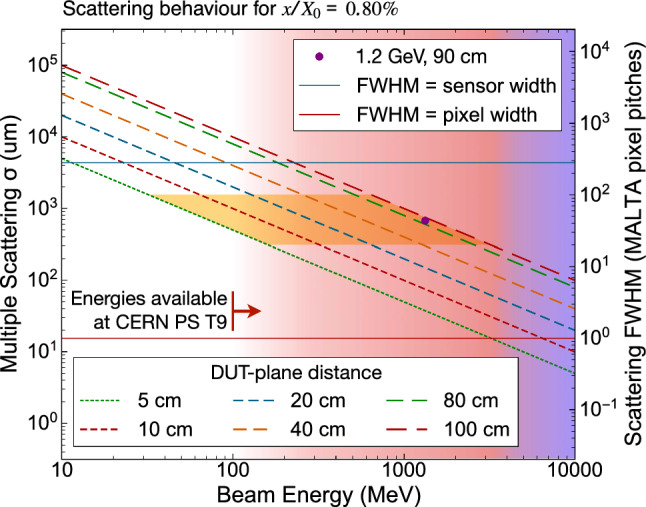
Relationship between beam energy and multiple scattering distribution width predicted for electrons/positrons for varying spacing distances between a subject with $x/X_0 = 0.8\%$ and a downstream plane at the given distance. The RMS of the scatter distribution $\sigma$, which equals $\theta ^{\text {RMS}}$ in Eq. 1, is shown in units of µm (left) and the FWHM of the distribution is shown in units of MALTA pixel pitches (right). The red and blue color backgrounds indicate the energy range where the PS T9 beam is dominated by positrons and pions, respectively. The intensity of the color is indicative for the expected rate. The horizontal blue line marks the upper limit where the scatter FWHM equals the sensor width, the horizontal red line marks the lower limit where the scatter FWHM equals the pixel pitch. The area highlighted in yellow indicates the expected telescope geometry and beam energy constraints for such measurements. The measurement presented in this paper is indicated as a violet point

Using Eq. 1, a relation between the beam energy and the width of the scatter angle distribution on a downstream plane at a given distance from a scattering target with thickness $x/X_{0}$ can be derived. This relationship is illustrated in Fig. 2 using both the standard deviation expressed in µm on the furthest downstream plane as well as the FWHM in units of pixel pitches of the telescope reference planes. For this comparison, the fractional radiation length of the device under test (DUT) was assumed to be $0.8\%$, corresponding to early estimates for the pixel module under test. The plot maps out the phase space to be considered for the measurement.

Non-hadronic beams are preferred for this measurement, to minimise the amount of hadronic interactions. Out of all beams that the T9 beam line at the CERN PS can provide, a $1.2\,$GeV positron beam was chosen for the main data-taking as it represented a compromise between purity, rate, and not requiring an unreasonably long telescope. It was configured with a beam momentum band of 5% FWHM, yielding approximately 6000 triggers per spill, with 2–3 spills per 40 s super-cycle, and representing a compromise between beam rate and an uncertainty on particle momenta derived from the momentum band.

**Fig. 3:**
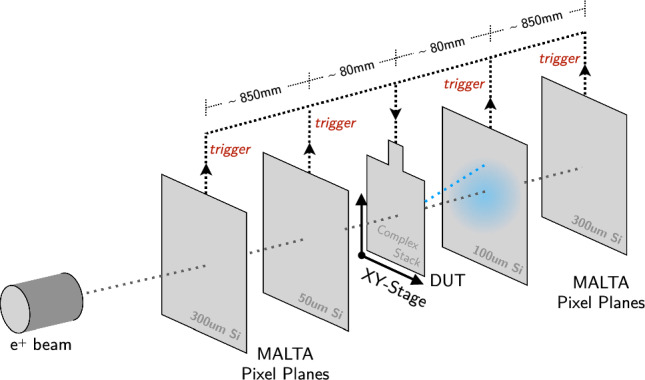
MONSTAR telescope configuration and trigger scheme, annotated with the plane thicknesses, and the spacing used. Multiple scattering within the DUT produces track kinks, which are analysed to derive the radiation length of the DUT

**Fig. 4:**
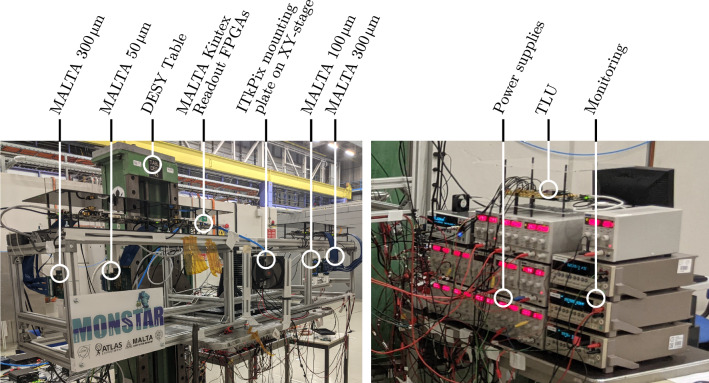
MONSTAR telescope setup during the CERN PS testbeam measurement

Following this choice, the MONSTAR telescope (Multiple Or Negligible Scattering Telescope, As Required) was constructed. It is a four plane telescope allowing the installation of a DUT on an $x-y$ linear stage as the central plane. The telescope frame is large enough to accomodate inter-plane spacing distances from about $5\,$cm to about $80\,$cm. The telescope setup is shown in Fig. 3. The reference planes contain depleted monolithic active pixel sensors from the MALTA Collaboration [15–18], with $512 \times 512$ pixels each and a pixel pitch of $36.4 \times 36.4\,$µm^2^. Two 300  µm thick Czochralski-type sensors [19] are used as outer planes, since they provide larger cluster sizes, and hence improved spatial resolution through use of a centre of gravity clustering algorithm. The inner two planes are 50  µm (upstream) and 100  µm (downstream) thick and contain less material to minimise the amount of multiple scattering within the telescope. Placed close to the DUT, these planes allow for long lever arm tracking without requiring data from the DUT itself, potentially allowing the telescope to be used for radiation length measurements on non-instrumented subjects. They are furthermore included in the trigger decision to reduce the rate of fake (noise) triggers. Trigger signals are generated from the coincidence of all four MALTA planes. An annotated photograph of the telescope is shown in Fig. 4. The readout system for the reference planes and the trigger logic unit (TLU) are loaded on Xilinx Kintex development boards and have been adopted from past MALTA beam telescopes [20].

**Fig. 5:**
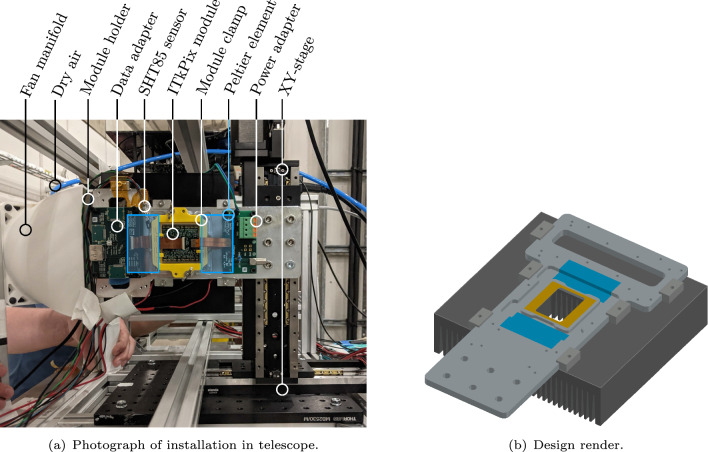
Custom mounting plate used to provide mechanics and cooling for the ITkPix module, annotated with the relevant components. The location of the Peltier elements between the holder and heatsink is denoted by the blue shaded regions. The thermal contact area between the module and holder is indicated by the orange shaded region. The window size beneath the module used during the measurement was $35 \times 25\,\text {mm}^2$, with the regions outside this window obscured by a thin ($450 \pm 3.9$) µm section of the aluminium holder

A pair of linear stages provide horizontal and vertical positioning capability of the DUT within the beam. A dedicated mounting plate was used to hold the ITk pixel module and provide cooling through the usage of two Peltier elements in combination with a heat sink and an air convection fan. The setup is shown in Fig. 5. A rectangular window in the mounting plate below the sensor ensures that the plate does not contribute to the material budget. Only a small fraction at the edges of the sensor is in physical contact with 450 µm thick aluminium to allow for sufficient heat transport to prevent the module from overheating.^2^ The physical contact is obtained through a 3D printed clamp that pushes the module on three edge points onto the aluminium. During operation, this cooling technique allowed to keep the module temperature constantly below $40\,^{\circ }\text {C}$ and hence ensure safe operation.

The ITk pixel module readout follows the same setup as the one foreseen in the actual detector. Therefore, this measurement also constitutes a readout test under realistic data taking conditions with external triggering. The signal is first transmitted via electrical links to an Optoboard [21] where it is converted to an optical signal fed into a readout server using FELIX [22, 23]. The readout software was integrated into the MALTA telescope software framework to allow for real time hit monitoring during data taking. Trigger input was accepted directly into the FELIX system as an LVDS signal from the MALTA TLU.

Data were taken using $1.2\,\text {GeV}$ positrons with a 5% momentum band, imaging the full $4 \times 4\,\text {cm}^2$ region of the ITk pixel module in 16 steps forming a $4\times 4$ grid of 1 cm pitch. The inner planes were moved close to the DUT to allow for tracking also without DUT hit information, with the spacings shown in Fig. 3. At least 2 million triggers were collected in each step, of which in general above 50% resulted in a well-reconstructed track within the $1\times 1\,\hbox {cm}^{2}$ region of interest. In addition, a reference dataset was taken with a 50 µm thick MALTA detector as DUT.

## Analysis

**Fig. 6:**
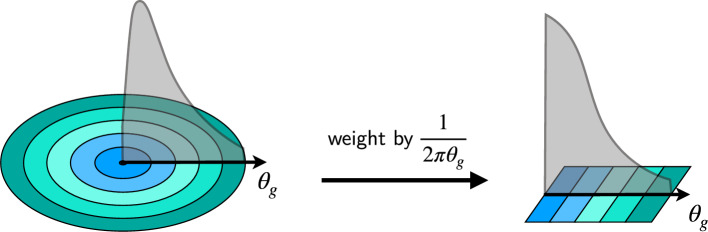
Transformation of the global angle $\theta _g$ to a pseudo-projected angle

A combination of the MALTA telescope software and the Corryvreckan testbeam analysis suite [24] is used for data quality checks and alignment, as well as tracking. A global frame is defined with the *z* axis pointing in beam direction and *x* and *y* chosen to align with the column and row directions of the DUT.

In all MALTA planes and the ITkPix DUT, noisy pixels are masked in each run separately using a local density method. If the pixel occupancy exceeds the local average by more than five standard deviations, it is masked and hits in this pixel are disregarded. A correction is added to the method to account for the higher occupancy of the central four rows and columns, which are double length to cover the inter-chip region on the quad module, and also exhibit high crosstalk. Trigger synchronisation is checked following a method described in Ref. [13] which relies on correlation of the *x* (and *y*) coordinates of hits across the different telescope planes in the synchronised case. No loss of synchronisation is observed for the data that were taken. Alignment is performed in two stages for each run, starting with a correlation-based pre-alignment followed by the application of the Millepede-II algorithm [25] to perform residual minimisation in *x*, *y* and $\theta _z$ (the plane rotation angle about the *z*-axis) for each plane, with all other degrees of freedom fixed.

Multiplet tracking is used to determine the incident and outgoing vectors from which the scatter angle is calculated. Separate straight line fits are performed to hits upstream and downstream of the DUT, with the DUT hit included in both the upstream and downstream tracklets unless otherwise specified. Accepted tracks are required to include one hit in every active plane. Uncertainties on the track origin and slope are computed from the linear fit, and propagated to the extracted angles between the track segments.

Multiplicative weight-based corrections are derived to correct for localised efficiency differences in the pixel detectors and geometric acceptance effects in the telescope. To minimise the geometric acceptance effects in the first place, the data-taking was divided into different segments with varied DUT positions making use of the linear stage. Residual acceptance effects are estimated by determining which fraction of tracks with an identical scatter angle would fall outside of the active area of any of the downstream planes. Over 96% of events intersecting the measurement region on the DUT were estimated to be in the acceptance of the downstream planes.

Two types of angles are extracted from the multiplet vectors and split into $\sim \,0.5\times 0.5\,\hbox {mm}^{2}$ subregions based on the scatter location:

1. Projected angles on the global *x*-*z* and *y*-*z* planes, $\theta _x$ and $\theta _y$.
2. Global $\phi$-invariant angles $\theta _g$, which are necessarily positive.

The projected angle distributions in each subregion are fit with a double-sided Crystal Ball function^3^ (DSCB) [26] in an unbinned negative log likelihood fit setup with RooFit [27]. The fitted width of the Gaussian core of the DSCB ($\sigma _{\theta }$) is then used as an estimator for $\theta ^{\text {RMS}}$, whilst the exponential tails are allowed to float to fit the $\sim 1/\sin ^4\theta$ single-scatter behaviour beyond the Gaussian core. Allowing the tails to float independently reduces bias from asymmetric distributions that can occur toward the edge of the measurement subject, where large scatters in one direction may leave the telescope acceptance at a higher rate than large scatters in the other direction.

**Fig. 7:**
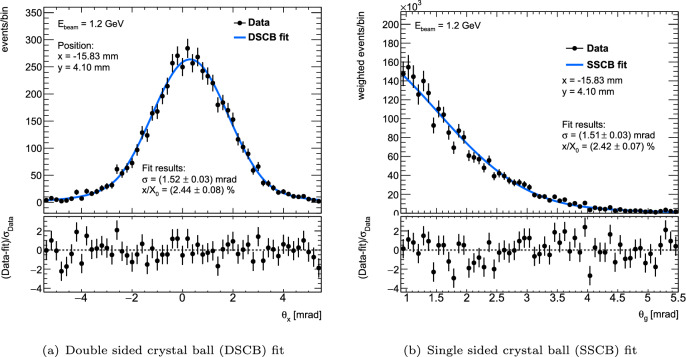
Example $\theta _x$ and $\theta _g$ distributions and fit functions for one subregion of one position of the DUT. The uncertainties on the quoted $x/X_0$ values include only statistical uncertainties

The global angle distribution is also considered and fit as a cross-check on the projected results, with the benefit of being independent of the definition of the *x*-*y* Cartesian reference frame defined by the projection planes. In order to treat the global angle distribution analogously to a projected distribution, each event is weighted by the inverse of the circumference of a circle with radius $\theta _g$. This procedure is sketched in Fig. 6. The resulting distribution is then fit using a single-sided Crystal Ball function (SSCB) with the fit range adjusted to remove events on the low side due to the large uncertainties caused by events being divided by small circumferences.^4^ Example fit results for both types of angles are shown in Fig. 7.

A model of the telescope has been simulated with the $\hbox {Allpix}^2$ framework [28] using the FTFP_BERT_EMZ GEANT4 physics list [14], which implements the most accurate step limit for multiple scattering for the given scatter energy. Running the full reconstruction chain on the simulated scatter events, the extracted $x/X_{0}$ values are compared to the values assumed in the simulation. This allows for a comparison of the extraction based on $\theta _x$ and $\theta _y$ with the extraction using a fit to $\theta _g$, in both cases using the inverse Highland formula. All three extraction methods agree within uncertainties, both with each other and with the $x/X_{0}$ value chosen in simulation. The $\theta _x$ and $\theta _y$ based extractions have been chosen as a baseline for the results shown in the following sections. Because $\theta _x$ and $\theta _y$ are by construction orthogonal, for the final results they are combined as individual measurements. A comparison with the $\theta _g$ based extraction is shown at the end of the paper in Fig. 12.

**Fig. 8:**
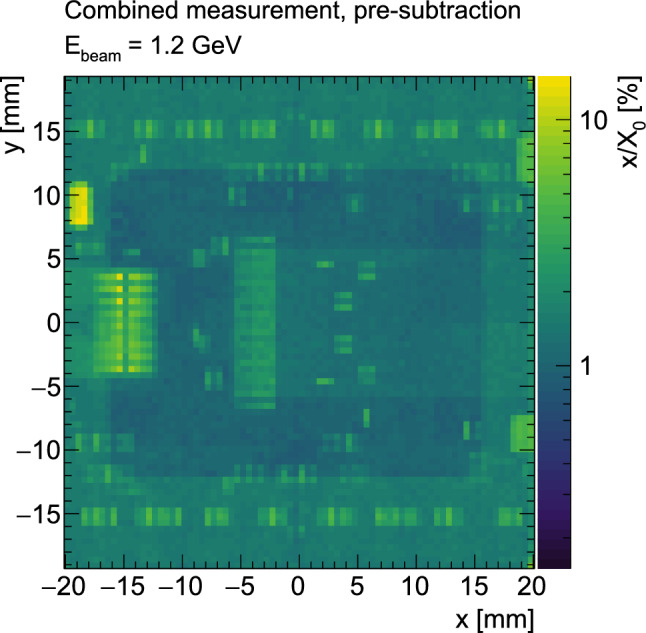
Measured radiation length $x/X_0$ for an ITkPix v1.1 pixel quad module. The radiation length is extracted from the multiple scattering angle of $1.2\,\text {GeV}$ positrons in the quad module using the inverse Highland formula. The radiation length map is shown before subtraction of the air and the module holder contributions

**Fig. 9:**
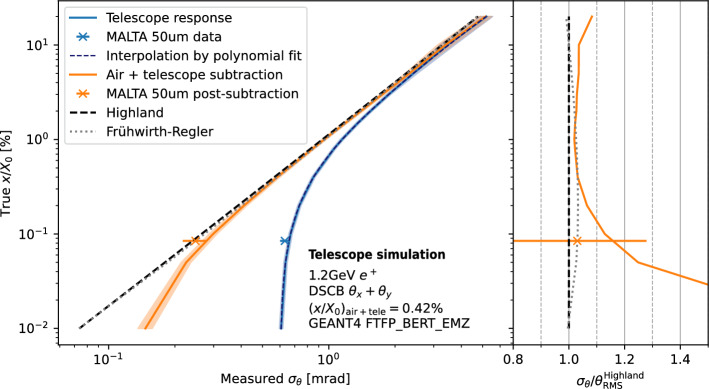
Simulation of the measured scatter angle RMS for various assumed true $x/X_0$ values of a homogeneous silicon DUT. The bands obtained from simulation are shown before (blue) and after the air + telescope subtraction (orange). The latter is compared to the to expectation from Highland (dashed line) and Frühwirth–Regler (dotted line). Both unsubtracted and subtracted simulations are compared to the reference measurement with a 50  µm thick MALTA plane as the DUT. On the left plot of the figure, the Highland and Frühwirth–Regler lines overlap. Their difference is only visible in the ratio plot on the right of the figure. A third-order polynomial fit to the response curve is shown (dark blue dashed line)

Figure 8 shows the fractional radiation length for the ITk pixel quad module under study extracted from the fitted $\theta _x$ and $\theta _y$ distributions. To arrive at the final measurement for the module, two residual corrections are made. First, the overlapping part of the aluminium frame from the module holder and the module clamp, which are both visible in Fig. 5, need to be subtracted. Second, the scatter contribution from the telescope reference planes as well as the air between the planes needs to be accounted for. The first subtraction is made based on radiation length estimates for aluminium and polylactide^5^ after measuring the thickness of the components. The telescope and air contribution is estimated from simulation and cross-checked with the reference measurement of the 50 µm thick MALTA detector as DUT, which is sufficiently homogeneous. The true material budget was estimated directly from the composition as $(x/X_0)_\textrm{MALTA}=0.53\%$. A comparison of the measurement results with the results obtained from simulation including the telescope and air contribution, as well as the agreement with the Highland and Frühwirth–Regler formulas after subtraction, is shown in Fig. 9. The reference measurement and the simulation agree within uncertainties, which validates the simulation. The telescope and air contribution is estimated from the measurement to $(x/X_0)_{\text {tel+air}} = (0.42 \pm 0.01)\%$. The final $x/X_{0}$ values are calculated as

$$\begin{aligned} \begin{aligned} \frac{x}{X_0}(x, y)&= \left( \frac{x}{X_0}(x, y)\right) _{\text {measured}} \\  &\quad - \left( \frac{x}{X_0}(x, y)\right) _{\text {module holder}}\\  &\quad - \left( \frac{x}{X_0}\right) _{\text {tel+air}} \quad \text {.} \end{aligned} \end{aligned}$$

## Results

**Fig. 10:**
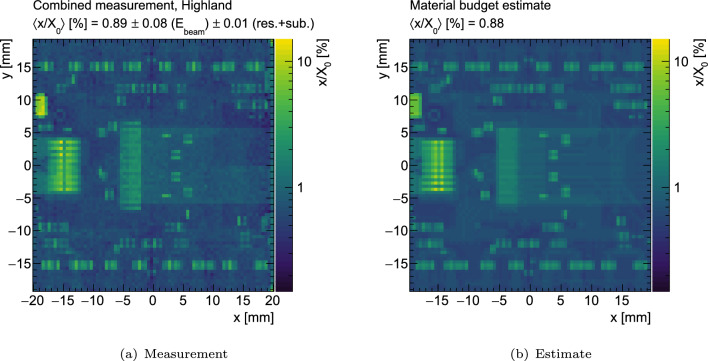
Measured and estimated fractional radiation length $x/X_0$ for an ITkPix v1.1 pixel quad module, populated with a 150  µm planar sensor and a quad flex v2.4. For the measured map, the inverse Highland formula has been used to convert the measured $\theta$ RMS values into $x/X_0$ values. The areas of largest radiation length correspond to the power and data connectors, the high voltage (HV) decoupling capacitor and the SMD components

**Fig. 11:**
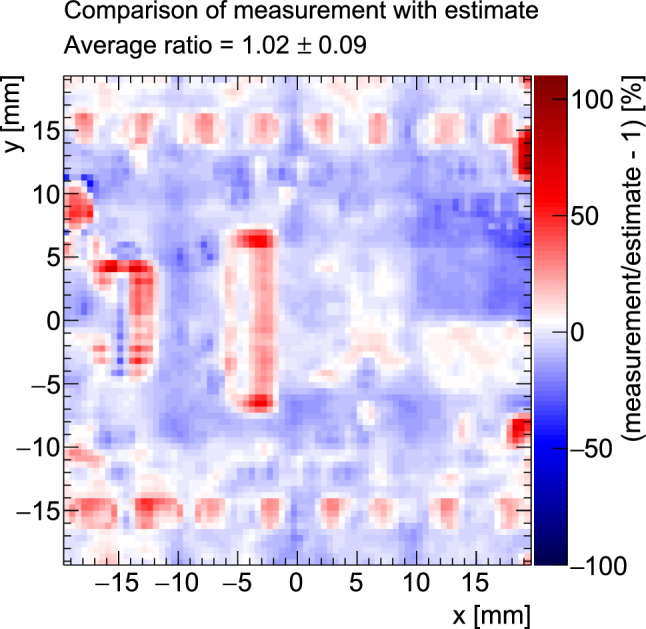
Comparison between the measured and expected radiation length maps. The largest differences are seen at the connectors and the SMD components, related to both their material budget as well as their placement on the PCB. Additionally, residual deviations from the subtraction of the support structures can be seen; in particular, deviations in the uniformity of the density of the 3D-printed clamp

The extracted $x/X_0$ map, based on the inverse Highland formula and post subtractions for the ITk pixel module under study is shown in Fig. 10. The areas of largest radiation length correspond to the power and data connectors, the high voltage (HV) decoupling capacitor and the SMD components, as illustrated in Fig. 1. Also shown in Fig. 10 is an independently derived estimate of the $x/X_0$ map that has been created based on the PCB design files and product data sheets for the SMD components and connectors, where available. Where not available, best guess values have been used. The average fractional radiation length of the module is measured to

$$\begin{aligned} \begin{aligned} \left\langle \frac{x}{X_0} \right\rangle _{\text {meas}} [\%] = 0.89&\pm 0.01~(\text {reso.})\\&\pm 0.01~(\text {subtraction})\\&\pm 0.08~(E_{\text {beam}}) \text {,} \end{aligned} \end{aligned}$$

which agrees with the estimate of $\langle x/X_0 \rangle _{\text {est}} = 0.88\%$. The largest source of uncertainty is the beam momentum band of $5\%$, which translates into a $9\%$ relative uncertainty on the extracted $x/X_0$ values after propagation through the inverse of the Highland formula. The statistical uncertainty on the average is negligible and about $0.05\%$ of a radiation length for each $0.5\times 0.5\,\hbox {mm}^{2}$ subregion of the map presented in Fig. 10. The uncertainty from the limited resolution of the telescope has been estimated by propagating a hit position uncertainty of $d/\sqrt{12}$ per plane, where *d* is the pixel pitch, to the scatter angles. This represents a conservative estimate for the resolution of each plane. Using varied toy datasets for the scatter angle distributions, the propagated resolution uncertainty is determined to be $0.01\%$ of a radiation length and therefore subdominant. The uncertainty on the subtraction is estimated from the uncertainty on the measurements or estimates of the individual components. This includes the aluminium module holder, the 3D-printed module holder clamp as well as the air subtraction. A spatially resolved comparison between estimate and measurement is shown in Fig. 11. Deviations of 50–100% are seen for regions including large material contributions that may not be well-modelled in the estimate, or may be misaligned with respect to the real module due to the pick-and-place accuracy during PCB population, such as proprietary connectors or surface-mount components on the module flex PCB.

**Fig. 12:**
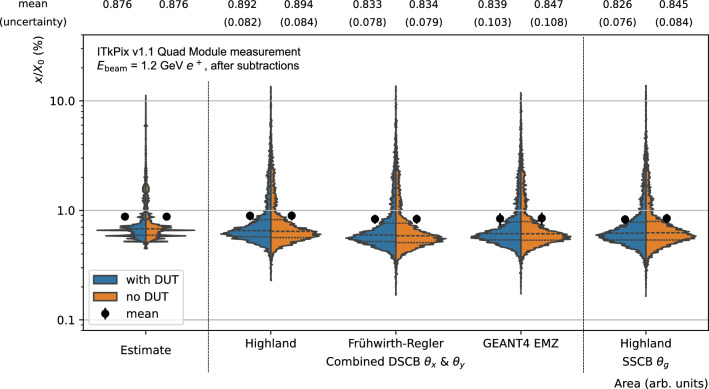
Violin plots comparing the estimated $x/X_0$ distribution of the ITk pixel module with three different extraction methods, based on the Highland formula, the Frühwirth–Regler formula and a GEANT4 based mapping of the measured $\theta$ to the underlying $x/X_0$ values. The dashed lines give quartiles of the distribution

While the baseline results in this paper have been obtained through a combination of fits to the projected scatter angles and using the inverse Highland formula, a comparison of all three extraction methods considered is shown in Fig. 12. In addition to the Highland and Frühwirth–Regler formalisms, a third method using the GEANT4-based simulation has been performed. This method uses simulated scatters to map the fitted $\sigma _{\theta }$ to the values of $x/X_{0}$ assumed in simulation. The simulated scatter target is assumed to be uniform silicon as the dominant material in the module material stack, and only the central momentum of the band is simulated. This response is then fitted with a third-order polynomial, as shown in Fig. 9. All three methods based on a combination of $\theta _x$ and $\theta _y$ fits agree well within uncertainties. The Highland formalism yields a 6–7% higher average $x/X_0$ compared to Frühwirth–Regler or the GEANT4-based extraction. In addition, for the extraction based on the Highland formalism, results obtained from a fit to the global scatter angle $\theta _g$ are shown in the same figure. They also agree with the aforementioned results and the estimate within uncertainties, confirming independence of the results with respect to the fit methodology.

The $x/X_0$ values are compared between the case with including the DUT hit in the fit (blue distributions) and the case without including it (orange distributions) and agree well. For the comparison without the DUT hit, the telescope and air subtraction based on the MALTA 50 µm reference has been repeated excluding the hit in the DUT. The subtraction without the DUT hit amounts to $(x/X_0)_{\text {tel+air}} = (0.46 \pm 0.01)\%$. The slight difference with respect to the subtraction value including the DUT hit suggests a non-trivial dependence of the air contribution on the tracking.

The estimated $x/X_{0}$ spectrum does not take into account the beam momentum spread and therefore contains large regions with identical radiation lengths, and in general a narrower spectrum than the measurement.

## Conclusion

A measurement of the fractional radiation length $x/X_0$ of an ATLAS ITk pixel module using multiple scattering of positrons in a low material beam telescope has been presented. The telescope has been built for the measurement and allows variable inter-plane spacings as well as *x*-*y* positioning of the DUT. Fitting kink angle distributions in regions of interest, an $x/X_0$ map with $\sim 0.5\,\text {mm}$ resolution and ${\mathcal {O}}(10\%)$ uncertainty has been derived. To calculate the $x/X_0$ values, methods based on the Highland and Frühwirth–Regler formalisms, and a simulation of the telescope with GEANT4 have been utilised and compared. Within the uncertainties of the measurement, all three methods agree with each other. The Highland formalism yields an average fractional radiation length of $(0.89 \pm 0.08)\%$ which agrees with an estimate of $0.88\%$, created from design drawings and component expectations.

The fact that the results do not change significantly when removing the DUT hit information from the kink angle extraction implies that this method can be applied to non-detecting materials as well. Future iterations of the measurement would benefit from a narrower beam momentum band as well as higher positron rates, to further increase the spatial resolution.

The achieved measurement uncertainty is of the same order as the current precision to which the ATLAS inner detector material budget is known [30], for some parts already better. The presented method could therefore be used to improve the knowledge of the material of certain components prior to physics data taking, ideally already in the R&D phase to influence the detector design.

## Data Availability

Data will be made available on reasonable request. [Author’s comment: The Corryvreckan testbeam framework is open-source software licensed under the MIT license and hence publicly available.]

## Footnotes

1 In the following referred to as the Highland formula for the sake of readability.

2 The exact dimensions of the window were optimised using finite element analysis simulations for the heat transport.

3 A double-sided Crystal Ball function is composed of a Gaussian distribution at the core, connected with two power law distributions describing the lower and upper tails.

4 The lower cut is determined by averaging the $0.5 \sigma$ values from the projected angle distributions $\theta _x$ and $\theta _y$.

5 Using the analytic formula given in Ref. [29] for $(\text {C}_{3}\text {H}_{4}\text {O}_{2})_{n}$.

## Code Availability Statement

Code/software will be made available on reasonable request. [Author’s comment: Auxiliary code produced as part of these studies will be made available on reasonable request.]
